# Supplementary material for: Transcriptome analysis indicates dominant effects on ribosome and mitochondrial function of a premature termination codon mutation in the zebrafish gene psen2
Source: PLoS One. 2020 Jul 13;15(7):e0232559. doi: 10.1371/journal.pone.0232559 (PMC7357760; doi:10.1371/journal.pone.0232559)
Supplement: S1 File — (PDF) [file pone.0232559.s001.pdf]

S1 Supplementary Data Files

S1 Appendix 1. ***psen2*-EGFP fusion gene sequences** as synthesized before cloning into the vector pcGlobin2

psen2WT-EGFP

HindIII BamHI *psen2* 5'UTR *psen2* codons 1-113 EGFP EcoRI

5′GACAAGCTTGGATCCTGGATTACCACGTGATGTGCTGGGGATCAGCTGATGGGGAATCACTTCCTC
ACTGAATTGGCGATGTTTTGACGGAGTATTTTCGCATAATACAGCGCTGGATCATCCTTCATGTAAAT
TCAGTCACATTTAAAGTTTCTCTTC**ATG**AATACCTCAGACAGTGAAGAGGACTCCTACAACGAGAGGT
CCGCTCTGGTCCAGTCCGAAAGCCCAACCGTGCCCTCATACAACCAAGACAATGCC**ATG**TCTCTCCCC
CAGGACACAGACAGCAAACGGAGCGGTGCGGTCCGCTCGAGGTCCGCCTCGGGCTCTGGAGATGCCGG
GCCGGTGGACAGAGAGCGCGCAGACACCCCTGATGGAGAGGAAGAGGAGCTCACACTCAAATACGGCG
CGAAACACGTCATC**ATG**CTCTTCATCCCTGTCACGCTCTGC**ATG**GTGGTCGTAGTGGCCACGATCAAG
TCCGTCAGTTTCTACACCGAGAAGGTGAGCAAGGGCGAGGAGCTGTTCACCGGGGTGGTGCCCATCCT
GGTCGAGCTGGACGGCGACGTAAACGGCCACAAGTTCAGCGTGTCCGGCGAGGGCGAGGGCGATGCCA
CCTACGGCAAGCTGACCCTGAAGTTCATCTGCACCACCGGCAAGCTGCCCGTGCCCTGGCCCACCCTC
GTGACCACCCTGACCTACGGCGTGCAGTGCTTCAGCCGCTACCCCGACCACATGAAGCAGCACGACTT
CTTCAAGTCCGCCATGCCCGAAGGCTACGTCCAGGAGCGCACCATCTTCTTCAAGGACGACGGCAACT
ACAAGACCCGCGCCGAGGTGAAGTTCGAGGGCGACACCCTGGTGAACCGCATCGAGCTGAAGGGCATC
GACTTCAAGGAGGACGGCAACATCCTGGGGCACAAGCTGGAGTACAACTACAACAGCCACAACGTCTA
TATCATGGCCGACAAGCAGAAGAACGGCATCAAGGTGAACTTCAAGATCCGCCACAACATCGAGGACG
GCAGCGTGCAGCTCGCCGACCACTACCAGCAGAACACCCCCATCGGCGACGGCCCCGTGCTGCTGCCC
GACAACCACTACCTGAGCACCCAGTCCGCCCTGAGCAAAGACCCCAACGAGAAGCGCGATCACATGGT
CCTGCTGGAGTTCGTGACCGCCGCCGGGATCACTCTCGGCATGGACGAGCTGTACAAGTAGGAATTC*A*
CG 3′

The *psen2* translation start codon S1 (ATG) is in bold, underlined text. Other possible downstream start codons (S2, S3, S4) are in bold text only. Black or blue text indicates successive *psen2* exon sequences.

psen2S4Ter-EGFP

HindIII BamHI *psen2* 5'UTR *psen2* codons 1-113 (with the 8bp sequence CAGACAGT deleted)EGFP EcoRI

5'GACAAGCTTGGATCCTGGATTACCACGTGATGTGCTGGGGATCAGCTGATGGGGAATCACTTCCTC
ACTGAATTGGCGATGTTTTGACGGAGTATTTTCGCATAATACAGCGCTGGATCATCCTTCATGTAAAT
TCAGTCACATTTAAAGTTTCTCTTC**<u>ATG</u>**AATACC**TGA**AGAGGACTCCTACAACGAGAGGTCCGCTCTG
GTCCAGTCCGAAAGCCCAACCGTGCCCTCATACAACCAAGACAATGCC**ATG**TCTCTCCCCCAGGACAC
AGACAGCAAACGGAGCGGTGCGGTCCGCTCGAGGTCCGCCTCGGGCTCTGGAGATGCCGGGCCGGTGG
ACAGAGAGCGCGCAGACACCCCTGATGGAGAGGAAGAGGAGCTCACACTCAAATACGGCGCGAAACAC
GTCATC**ATG**CTCTTCATCCCTGTCACGCTCTGC**ATG**GTGGTCGTAGTGGCCACGATCAAGTCCGTCAG
TTTCTACACCGAGAAGGTGAGCAAGGGCGAGGAGCTGTTCACCGGGGTGGTGCCCATCCTGGTCGAGC
TGGACGGCGACGTAAACGGCCACAAGTTCAGCGTGTCCGGCGAGGGCGAGGGCGATGCCACCTACGGC
AAGCTGACCCTGAAGTTCATCTGCACCACCGGCAAGCTGCCCGTGCCCTGGCCCACCCTCGTGACCAC
CCTGACCTACGGCGTGCAGTGCTTCAGCCGCTACCCCGACCACATGAAGCAGCACGACTTCTTCAAGT
CCGCCATGCCCGAAGGCTACGTCCAGGAGCGCACCATCTTCTTCAAGGACGACGGCAACTACAAGACC
CGCGCCGAGGTGAAGTTCGAGGGCGACACCCTGGTGAACCGCATCGAGCTGAAGGGCATCGACTTCAA
GGAGGACGGCAACATCCTGGGGCACAAGCTGGAGTACAACTACAACAGCCACAACGTCTATATCATGG
CCGACAAGCAGAAGAACGGCATCAAGGTGAACTTCAAGATCCGCCACAACATCGAGGACGGCAGCGTG
CAGCTCGCCGACCACTACCAGCAGAACACCCCCATCGGCGACGGCCCCGTGCTGCTGCCCGACAACCA
CTACCTGAGCACCCAGTCCGCCCTGAGCAAAGACCCCAACGAGAAGCGCGATCACATGGTCCTGCTGG
AGTTCGTGACCGCCGCCGGGATCACTCTCGGCATGGACGAGCTGTACAAGTAGGAATTC*ACG* 3'

The *psen2* translation start codon S1 (ATG) is in bold, underlined text. Other possible downstream start codons (S2, S3, S4) are in bold text only. Black or blue text indicates successive *psen2* exon sequences. The putative translation stop codon (TGA) generated by the *S4Ter* mutation downstream of S1 is shown in red text.

<u>Predicted fusion protein masses for translation beginning at each potential start codon S1-S4</u>:

S1, 39 kDa
S2, 35 kDa
S3, 29 kDa
S4, 28 kDa

EGFP alone, 25 kDA

S1 Table 1. Expression levels of the wild type *psen2* allele in 25ng total adult brain cDNA

Numbers represent cDNA copy numbers detected on the QuantStudio™ 3D Digital PCR 20K Chip after processing by QuantStudio™ 3D AnalysisSuite Cloud Software (Life Sciences, Waltham, MA, USA).

**+/+ zebrafish under normoxia**	***psen2^{S4Ter}*/+ zebrafish under normoxia**	***psen2^{S4Ter}*/*psen2^{S4Ter}* zebrafish under normoxia**
1241.5	696.48	2.538
1116.2	658.88	2.765
1539.4	793.29	3.445

**+/+ zebrafish under acute hypoxia**	***psen2^{S4Ter}*/+ fish under acute hypoxia**	***psen2^{S4Ter}*/*psen2^{S4Ter}* zebrafish under acute hypoxia**
1739.8	893.43	2.862
1667.6	837.51	3.562
1925.9	1280.4	0.966

S1 Table 2. Expression levels of the *psen2*^{S4Ter} allele in 25ng total adult brain cDNA

Numbers represent cDNA copy numbers detected on the QuantStudio™ 3D Digital PCR 20K Chip after processing by QuantStudio™ 3D AnalysisSuite Cloud Software (Life Sciences, Waltham, MA, USA).

**+/+ zebrafish under normoxia**	***psen2*^{S4Ter}/+ zebrafish under normoxia**	***psen2*^{S4Ter}/*psen2*^{S4Ter} zebrafish under normoxia**
0.11	818.48	832.91
0.364	561.92	1144.2
0.108	655.45	1204.1

**+/+ zebrafish under acute hypoxia**	***psen2*^{S4Ter}/+ zebrafish under acute hypoxia**	***psen2*^{S4Ter}/*psen2*^{S4Ter} zebrafish under acute hypoxia**
5.432	940.09	1721.5
4.297	855.15	2048.8
4.732	1289.4	1931.8

S1 Table 3. Numbers of DoLA neurons in 24 hpf embryos (revealed by *in situ* transcript hybridization against *tbx16* mRNA)

The number in each cell represents the number of DoLA neurons counted in an individual embryo

+/+	*psen2*^{S4Ter}/+	*psen2*^{S4Ter}/*psen2*^{S4Ter}
20	16	19
23	9	20
22	17	18
17	23	27
17	21	22
	18	23
	22	20
	26	17
	24	27
	23	23
	26	17
	23	18
	18	22
	14	20
	17	20
	16	24
	23	
	17	
	17	
	17	
	16	

S1 Appendix 2. *psen2^{S4Ter}* transcript splicing tests

The *psen2^{S4Ter}* allele has an 8 bp deletion in zebrafish *psen2* exon 2 causing a frame shift leading to a premature termination codon. To test whether this mutation affects transcript splicing (possibly skipping the premature termination/stop codon) we performed PCR on cDNA derived from wild type or homozygous mutant 24 hpf embryos.

The diagram below shows the oligonucleotide primer pairs used in the PCRs and their binding sites in *psen2* exons.

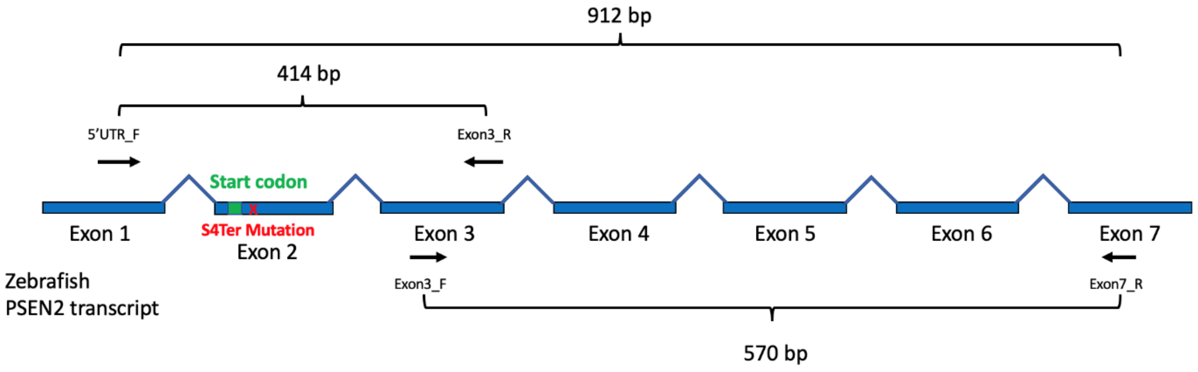

The exons shown above have the following ID codes in Ensembl based on transcript psen2-201 with Ensembl ID: ENSDART00000006381.8:

***Psen2* exon number**	**Ensembl ID**
1	ENSDARE00000480737
2	ENSDARE00000116019
3	ENSDARE00000115815
4	ENSDARE00000014141
5	ENSDARE00000101632
6	ENSDARE00000089891
7	ENSDARE00000961856

**PCR Primer Name**	**Sequence**
5'UTR_F	5'- TTTTGACGGAGTATTTTCGCAT-3'
Exon3_F	5'- CTCTTCATCCCTGTCACGCTCT-3'
Exon3_R	5'- CTCGGTGTAGAAACTGACGGACTT-3'
Exon7_R	5'- TTCCACCAGCATCCTCAACG-3'

RNA purification, cDNA preparation and PCR conditions are described in the main paper.

PCR Results

"*Psen2* 5'UTR(exon1)-exon3" used primer 5'UTR_F paired with Exon3_R.

"*Psen2* exon3-exon7" used primer Exon3_F paired with Exon7_R.

"*Psen2* 5'UTR(exon1)-exon7" used primer 5'UTR_F paired with Exon7_R.

PCR products were electrophoresed through a 1.5% agarose gel in 1×TAE buffer for separation and identification.

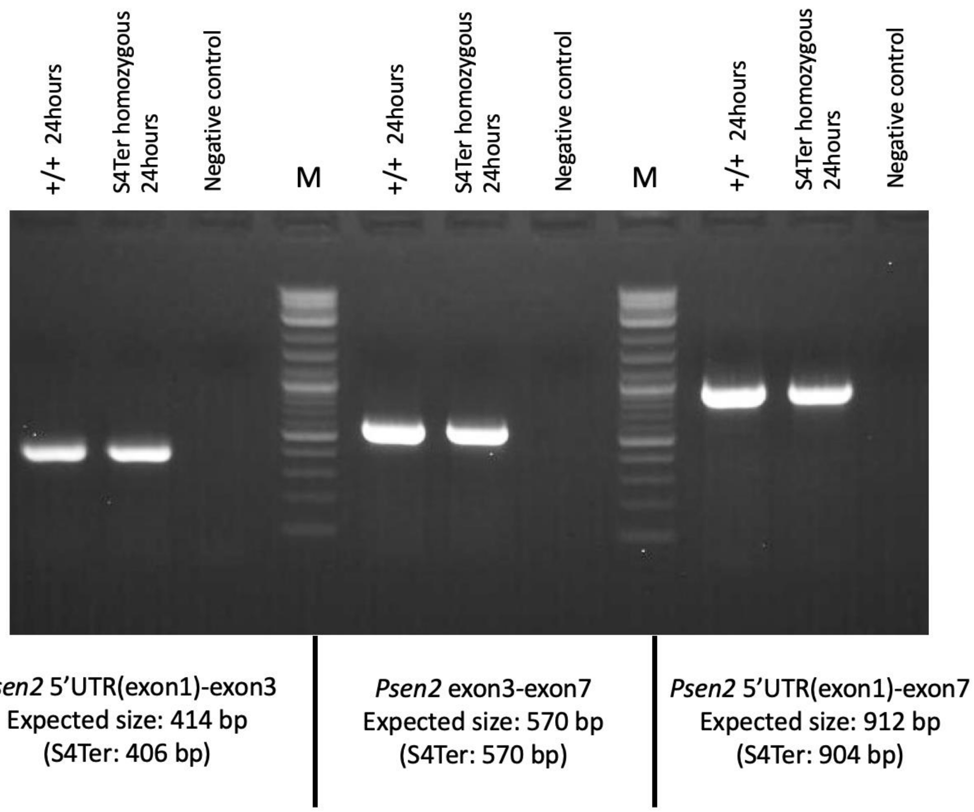

In all three PCR tests, no differences in product (band) sizes are observed for cDNA derived from wild type and *psen2*^{S4Ter} homozygous embryos. Thus we did not detect aberrant transcript splicing in the mutants.
