# Supplementary material for: Transcriptome analysis indicates dominant effects on ribosome and mitochondrial function of a premature termination codon mutation in the zebrafish gene psen2
Source: PLoS One. 2020 Jul 13;15(7):e0232559. doi: 10.1371/journal.pone.0232559 (PMC7357760; doi:10.1371/journal.pone.0232559)

S3 Raw Images Supplementary Data File.

Raw images of western immunoblotting

The raw images used to assemble Fig 4B are shown below. “X” marks blot lanes not included in Fig 4B. Bound antibody was detected by chemiluminescence using SuperSignal™ West Pico PLUS Chemiluminescent Substrate (Thermo Fisher Scientific) and imaged by the ChemiDoc™ MP Imaging System (Bio-Rad Laboratories).

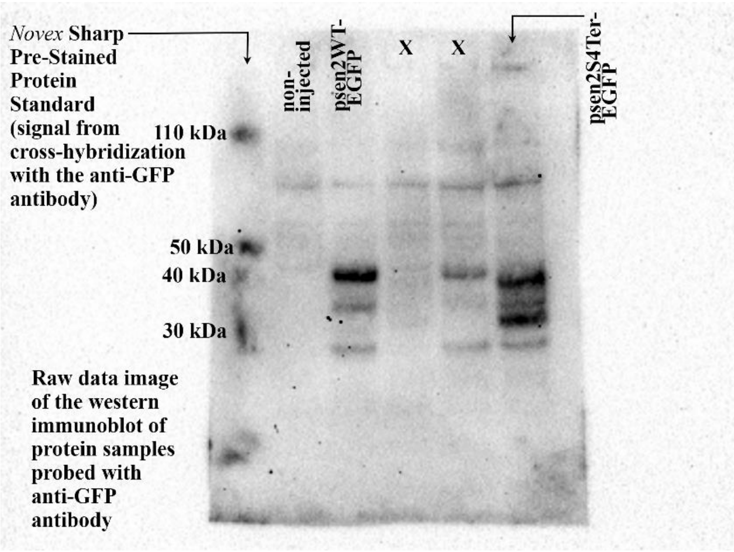

After imaging of protein detected by the anti-GFP antibody, this was stripped from the blot and then the blot was probed with an antibody against Tubulin as a loading control. Some faint bands not consistent with the presence of Tubulin are present where removal of the previous antibody from the blot was incomplete:

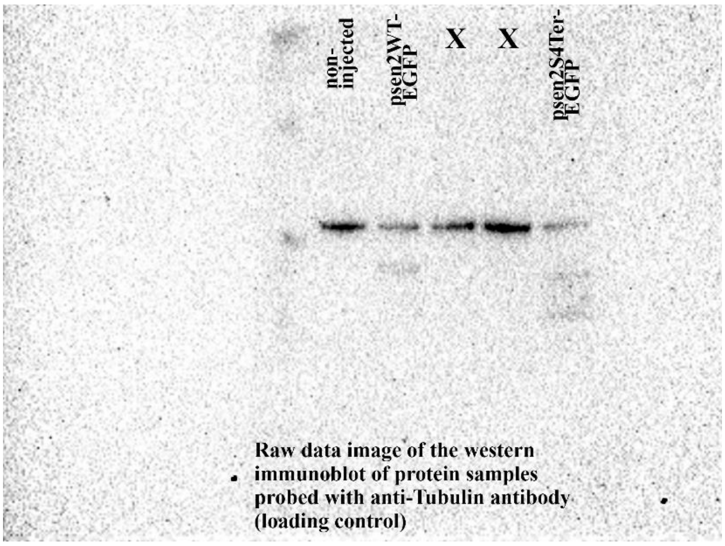

Supplement: S1 Raw images — (PDF) [file pone.0232559.s003.pdf]
